# Ehlers–Danlos Syndrome: Not Just Joint Hypermobility

**DOI:** 10.1155/2018/5053825

**Published:** 2018-08-29

**Authors:** Tina Bregant, Milica Klopcic Spevak

**Affiliations:** University Rehabilitation Institute of Republic of Slovenia, Linhartova 51, 1000 Ljubljana, Slovenia

## Abstract

Ehlers–Danlos syndrome is an umbrella term for a group of heritable soft connective tissue disorders which is characterized by joint hypermobility, skin texture and elasticity abnormalities, and visceral and vascular fragility or dysfunctions. As the syndrome is rare, it is often underdiagnosed. Patients usually present late, with chronic moderate to severe pain which is attributed to the joint hypermobility and joint subluxations. If the clinician is aware of the syndrome, he/she can identify affected patients in order to prevent complications. We report a 60-year-old woman with arthralgia and back pain lasting for several months and recent metatarsophalangeal luxation of the left toe who was discovered to have Ehlers–Danlos syndrome.

## 1. Introduction

Ehlers–Danlos syndrome (EDS) is an umbrella term for a group comprising heritable soft connective tissue disorders characterized by generalized joint hypermobility, skin texture abnormalities, and visceral and vascular fragility or dysfunctions [1]. Major and minor diagnostic criteria have been defined for each type and complemented whenever possible with laboratory findings; the clinical Villefranche classification identifies six major EDS variants [1]. Advances in molecular testing have made it possible to identify the causative mutation in the majority of patients showing that abnormalities in type V collagen are the cause of a classical type EDS [2].

There is a clinical overlap with the joint hypermobility syndrome (JHS) [3]. Joint hypermobility is relatively abundant, found in up to one-third (30%) of children [4]. It can present in a variety of signs and symptoms such as musculoskeletal and neuropathic pain, joint instability, fatigue, anxiety, and cardiovascular problems [5–7].

Adults diagnosed with JHS/EDS-hypermobile type often experience joint pain or widespread pain of different levels of severity [8]. In each affected individual, clinical decision-making should be theoretically applied for now, underpinned by the available evidence, making each of the patients with EDS unique in a therapeutic approach.

## 2. Case Presentation

A 60-year-old woman with arthralgia and back pain lasting for several months and recent metatarsophalangeal luxation of the left toe presented to the ambulatory unit of rehabilitation clinics. She was referred to the clinics as a patient with chronic pain syndrome. The patient had joint hypermobility since childhood, diagnosed as ligament laxity. In adolescence, she is remembered to be called “a clumsy freak” due to joint mobility.

**Figure 1:**
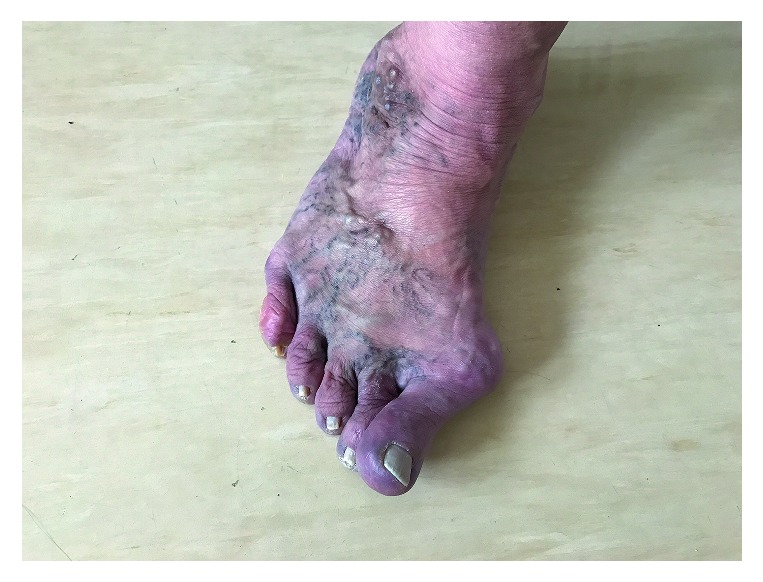
Hallux valgus and subluxation of the proximal interphalangeal (PIP) joint of the toes, varicose veins, and paperlike skin in our patient.

At physical evaluation, marfanoid habitus with waxy, sagging skin and varicose veins in the feet was observed. Sclerae were bluish and eyelids dropping. In fingers and toes, spontaneous subluxation in all joints could be elicited. Elbows, knees, and all fingers were overextended. She had flat feet with a bilateral hallux valgus (Figure 1). She had scoliosis with prominent kyphosis. Lungs auscultation was characteristic of chronic obstructive pulmonary disease (COPD); the murmur of mitral valve prolapse was heard over the chest.

**Figure 2:**
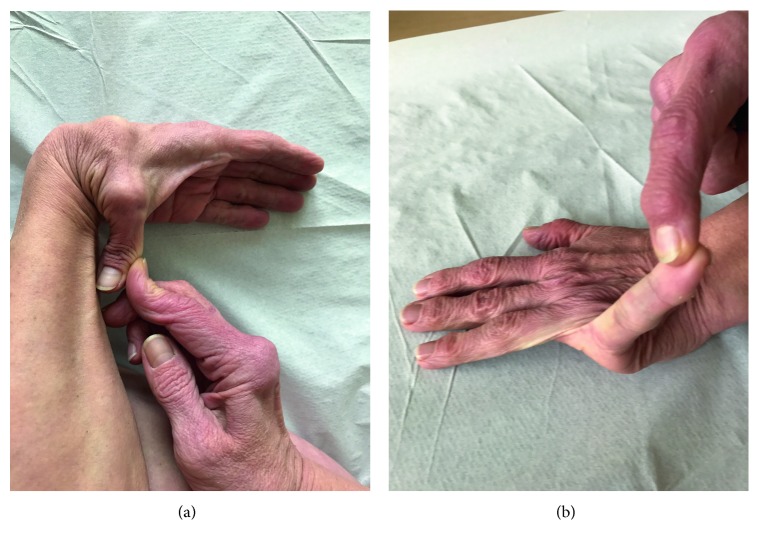
Joint hypermobility: passive apposition of the thumb to forearm and passive dorsal hyperextension of the metacarpophalangeal joint >90° (Beighton score: questions 1 and 2).

On the Beighton score, she received all (9) scores: passive apposition of the thumb to forearm and passive dorsal hyperextension of the metacarpophalangeal joint >90° on both sides were done with no strain (Figure 2); she was able to actively hyperextend both elbows and knees on both sides over >10° and flex her spine to the ground with palms placed on the ground without knee flexing. On Five-point Hypermobility Questionnaire, she answered “yes” to all questions. She remembered vividly contorting her body into strange shapes and being called names by other children. Genetic analysis showed a typical mutation consistent with the classical Ehlers–Danlos syndrome.

In the ambulatory unit, she received systematic, light, nonweightbearing, and proprioception exercises; she was referred to the occupational therapist for lower limb orthosis; she was taught relaxation techniques including mindfulness-based stress reduction and counselling support, though she was already familiar with cognitive behavioural therapy. She used regular anti-inflammatory drugs. She was referred to the plastic surgeon due to the wound on her foot, since sutures should be applied generously, without tension, in layers, and left in place twice as long as usual. She was already regularly followed by her cardiologist due to mitral valve prolapse.

## 3. Discussion

In our patient, EDS was recognized late. This was due to attributing problems of hypermobility only.

Pain is common in patients with EDS and may correlate with hypermobility, frequency of subluxations and dislocations, soft tissue injury, history of previous surgery, and myalgias and may become chronic [9]. Response to traditional pain medication is not adequate probably because the underlying cause is different to most other pains [10]. That is why the therapy should aim not only into pain management but also into the management of fatigue, dystonia, energy consumption, and the treatment of the impaired proprioception among others [10]. Earlier diagnosis may avoid long diagnostic and therapeutic paths focusing on the other aspects of the syndrome including screening for cardiovascular complications instead of focusing on chronic pain only.

## 4. Conclusions

Ehlers–Danlos syndrome is a rare disease of the connective tissue and a diagnostic challenge. Late diagnosis leads to chronic pain and complications, limiting daily activity and mobility. This case report serves to remind the rehabilitation clinicians that it is important to identify all affected patients in order to prevent complications instead of just providing the therapy against pain.

## References

[1] Beighton P., De Paepe A., Steinmann B., Tsipouras P., Wenstrup R. J. (1998). Ehlers-Danlos syndromes: revised nosology, Villefranche. *American Journal of Medical Genetics*.

[2] Bowen J. M., Sobey G. J., Burrows N. P. (2017). Ehlers–Danlos syndrome, classical type. *American Journal of Medical Genetics Part C (Seminars in Medical Genetics)*.

[3] Tinkle B. T., Bird H. A., Grahame R., Lavallee M., Levy H. P., Sillence D. (2009). The lack of clinical distinction between the hypermobility type of Ehlers–Danlos syndrome and the joint hypermobility syndrome (a.k.a. hypermobility syndrome). *American Journal of Medical Genetics Part A (Seminars in Medical Genetics)*.

[4] Simmonds J. V., Keer R. J. (2007). Hypermobility and the hypermobility syndrome. *Manual Therapy*.

[5] Bulbena A., Gago J., Pailhez G., Sperry L., Fullana M. A., Vilarroya O. (2011). Joint hypermobility syndrome is a risk factor trait for anxiety disorders: a 15-year follow-up cohort study. *General Hospital Psychiatry*.

[6] Smith T. O., Easton V., Bacon H. (2014). The relationship between benign joint hypermobility syndrome and psychological distress: a systematic review and meta-analysis. *Rheumatology*.

[7] Hakim A. J., Grahame R. (2004). Non-musculoskeletal symptoms in joint hypermobility syndrome. Indirect Evidence for autonomic dysfunction?. *Rheumatology*.

[8] Engelbert R. H., Juul-Kristensen B., Pacey V. (2017). The evidence-based rationale for physical therapy treatment of children, adolescents, and adults diagnosed with joint hypermobility syndrome/hypermobile Ehlers–Danlos syndrome. *American Journal of Medical Genetics Part C (Seminars in Medical Genetics)*.

[9] Mulvey M. R., Macfarlane G. J., Beasley M. (2013). Modest association of joint hypermobility with disabling and limiting musculoskeletal pain: results for a largescale general population-based survey. *Arthritis Care and Research*.

[10] Chopra P., Tinkle B., Hamonet C. (2017). Pain management in the Ehlers–Danlos syndromes. *American Journal of Medical Genetics Part C (Seminars in Medical Genetics)*.

